# Importance of clinico‐pathologic correlation in rare, chronic infectious diseases: Actinomycetoma misdiagnosed as botryomycosis—A case report

**DOI:** 10.1002/ccr3.8977

**Published:** 2024-05-23

**Authors:** Josiah Tatenda Masuka, Luanda Mthembu, Khumo Duze, Ameshin Moodley, Tshikani Norman Rikhotso, Anisa Mosam

**Affiliations:** ^1^ Department of Dermatology, Nelson Mandela School of Medicine University of KwaZulu‐Natal Durban South Africa; ^2^ Department of Dermatology Inkosi Albert‐Luthuli Central Hospital Durban KwaZulu‐Natal South Africa; ^3^ Department of Medicine, Parirenyatwa Building, Faculty of Health Sciences University of Zimbabwe Mazowe Zimbabwe; ^4^ Department of Pathology Inkosi Albert‐Luthuli Central Hospital Durban KwaZulu‐Natal South Africa

**Keywords:** actinomycosis, botryomycosis, clinical challenges, diagnostic errors, differential diagnosis, Madura foot

## Abstract

This case report explores the clinical journey of a patient initially diagnosed with botryomycosis, only to later reveal the underlying and rare condition of actinomycosis. The report highlights the challenges in getting to an accurate diagnosis, emphasizing the importance of considering uncommon pathologies, the utility of multi‐disciplinary teams and clinico‐pathologic correlation in clinical practice.

## Graphical abstract

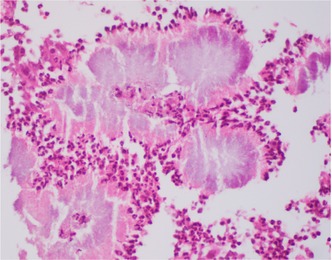

## 1 INTRODUCTION

The Madura foot or mycetoma is a localized, chronic and progressively destructive granulomatous infection of the subcutaneous tissues that spreads to the skin, deep tissues, and bone.^1^, ^2^ Myecetoma is a World Health Organization‐designated neglected tropical disease commonly reported in patients from low socio‐economic backgrounds in the “Myectoma belt.”^2^, ^3^ Although there is uncertainty to the actual global burden of mycetoma, an estimated prevalence of 14.5% per 1000 population has been recorded in Sudan, one of the most endemic countries for the disease in the world.^3^

Mycetoma develops after inoculation of the subcutaneous tissues with saprophytic fungal or filamentous bacterial organisms from the soil or vegetable matter following a penetrating skin injury.^1^ It commonly affects the limbs, especially the foot, and was first described in Madurai, India, hence the term “Madura foot.”^1^, ^4^ Mycetoma results from either true fungi (eumycetoma) or Gram‐positive aerobic bacteria (actinomycetoma),^2^ accounting for 40% and 60% of cases, respectively.^1^ Classically both eumycetoma and actinomycetoma present with a triad of painless subcutaneous swelling, multiple draining sinuses whose discharge contains grains.^5^ Histologically, both also show the Splendori–Hoeppli phenomenon or reaction,^6^ thus eumycetoma and mycetoma may not easily be distinguished. Clinical diagnosis is further complicated by a wide differential diagnosis for both these clinical and histopathologic findings with mimickers such as cutaneous botryomycosis^3^, ^6^, ^7^ which present with similar clinical,^3^, ^8^, ^9^ radiological and histologic findings,^6^, ^7^, ^9^ thus confounding the diagnostic process. Here we present a challenging case with both clinical and laboratory findings suggestive of cutaneous botryomycosis, but only responsive to therapy for actinomycetoma.

## 2 CASE REPORT

**FIGURE 1:**
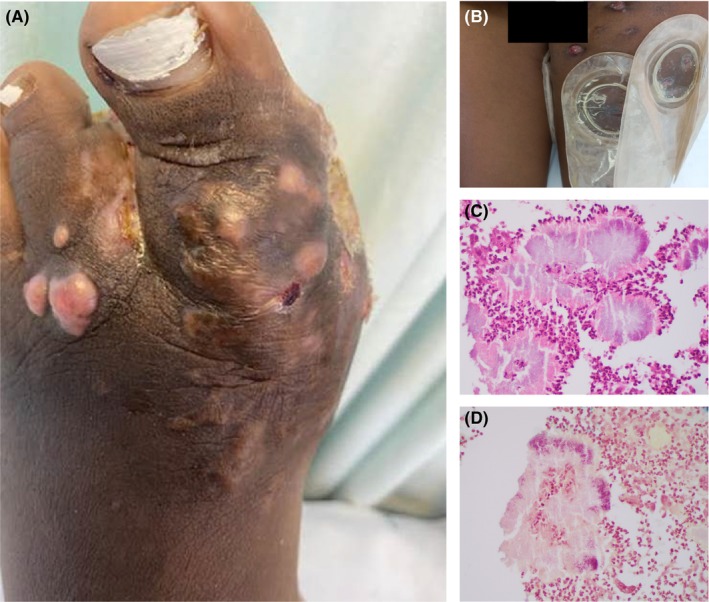
Pre‐treatment clinical and histology pictures. The foot nodules (A) and the swollen thigh with draining sinuses (B); the Splendori–Hoeppli phenomenon (H&E stain ×40 magnification) (C) and the filamentous bacteria (Brown‐Hopps stain ×40 magnification) (D).

A 23‐year‐old female patient presented to our dermatology clinic with a 4‐year history of painless, slowly growing, nodular lesions on the left foot associated with unilateral thigh swelling, draining sinuses and groin lymphadenopathy as shown in Figure 1A,B. The referring general surgeons had suspected Kaposi sarcoma and biopsied the lesions. On presentation to dermatology, the surgeons' biopsy showed a dermal granulomatous inflammation demonstrating the Splendore–Hoeppli phenomenon highlighted in Figure 1C,D. Her past medical and surgical history was unremarkable, and she was of sober habits. She had microcytic anemia as shown in her laboratory results in Table 1. Based on these findings and the subsequent tissue and aspirate culture results which persistently showed *Methicillin Resistant Staphylococcus Aureus* (*MRSA*) infection, we made a diagnosis of cutaneous botryomycosis and treatment with cotrimoxazole 2 tablets twice daily, orally was initiated. On noticing a lack of response, the cotrimoxazole therapy was replaced with *MRSA* sensitivity‐directed therapy with vancomycin 1 g twice daily. Despite 2 months on the initial treatment, the patient's disease progressed, indicated by persistent nodules, thigh swelling, sinuses' discharge, and an associated anemia (Hb 5 g/dL).

**TABLE 1:** Laboratory findings.

Test	Parameter	Value	Range
Full blood count	White cell count/×10^9^ cells/L	12.94	3.92–10.40
	Hemoglobin/g/dL	6.6	13.0–17.0
	Mean corpuscular volume/fL	78.1	78.9–98.3
	Mean corpuscular hemoglobin/pg	23.3	26.1–33.5
	Platelets /×10^9^ cells/L	517	171–388
Differential count	Neutrophils/×10^9^ cells/L	10.05	1.61–8.30
	Lymphocytes/×10^9^ cells/L	2.14	1.60–4.50
Urea and eletrolytes and liver function tests		Normal	

## 3 METHODS (DIFFERENTIAL DIAGNOSIS, INVESTIGATIONS, AND TREATMENT)

A re‐evaluation of the clinical presentation, histology and medical microbiology findings was carried out. We now suspected that the diagnosis was actinomycetoma though this was difficult since the patient only had nodules and sinuses and no grains had been observed. Additional laboratory investigations showed negative HIV serology and a normal lymphocyte flow cytometry. Radiological investigations showed a hematoma on ultrasound scan (USS) of the affected limb's thigh with no lytic bone lesions on X‐ray. Computed tomography (CT) additionally showed mural enhancement of popliteal and superficial femoral veins and multiple ring‐enhancing collections, suggestive of phlebitis and an infective process, respectively. Upon discussion with histopathologists and medical microbiologists, a re‐biopsy was ordered on deeper sections of the foot lesions as well as a nasal swab. While awaiting the results of these tests, the patient was started on a trial of the Welsh regimen, consisting of an intensive phase of amikacin 15 mg/kg/day intravenously, 12 hourly for 21 days (1 cycle), 3 cycles with an interval of 15 days and a maintenance course of cotrimoxazole (trimethoprim–sulfamethoxazole) 320/1200 mg twice daily orally for 6 months.^10^

## 4 OUTCOME AND FOLLOW‐UP

**FIGURE 2:**
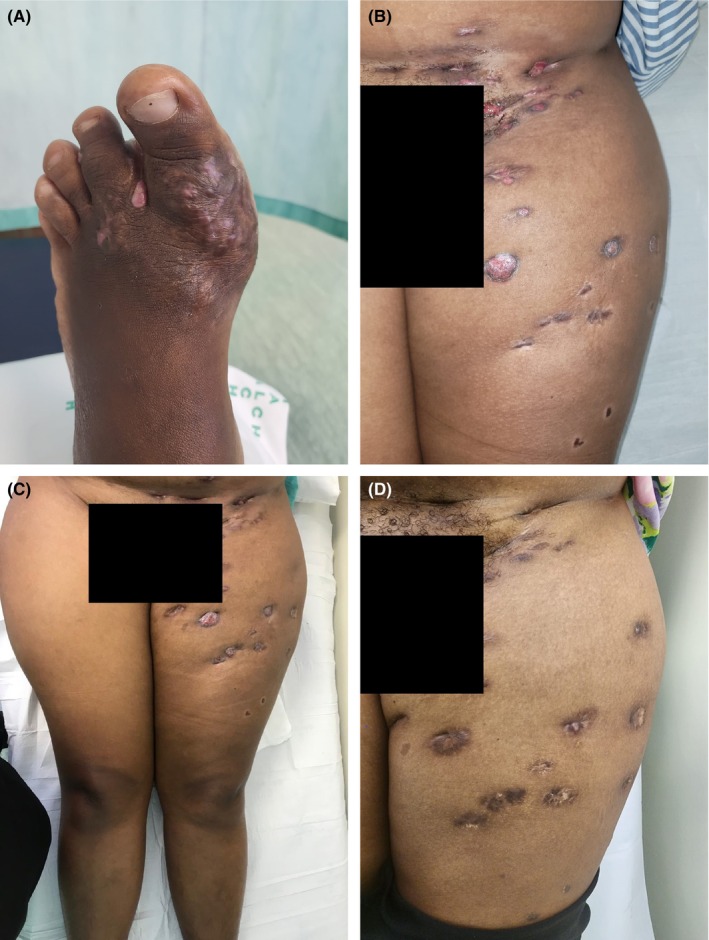
Clinical pictures 5 months post‐initiation of the Welsh regimen demonstrating marked improvement on the foot (A) and (B) and persistent sinuses (C), while complete resolution of the thigh lesions is shown in (D) at the completion of treatment.

The results of the re‐biopsy of the foot lesions, subsequently revealed Gram‐positive filamentous bacteria, lacking Ziehl Nielsen or Fite staining. Thus, confirming a diagnosis of actinomycotic mycetoma. Concurrently, a nasal swab showed nasal carriage of staphylococcus aureus, thereby explaining the persistently cultured staphylococcus bacteria as a super infection. While on the Welsh regimen, the patient started showing remarkable clinical improvement with disappearance of the cutaneous nodules, cessation of the thigh sinus discharge, and resolution of anemia. Her foot lesions resolved first, around 5 months after initiating the amikacin and sulfamethoxazole‐trimethoprim regimen while the thigh lesions persisted as shown in Figure 2A–C. They only resolved after 10 completed months of dosing with the Welsh regimen as shown in Figure 2D.

## 5 DISCUSSION

We have reported a case of actinomycetoma initially misdiagnosed as cutaneous botryomycosis. The patient presented to us with a swollen foot and draining sinuses associated with laboratory findings indicating *MRSA* infection on bacterial culture and demonstrating the Splendori–Hoeppli reaction on biopsy. However, on failing to respond to apparently adequate doses of cotrimoxazole and later drug sensitivity directed vancomycin antibiotic therapy,^11^ the case was re‐evaluated. Only after constituting a multi‐disciplinary team involving dermatologists, microbiologists, and histopathologists, a correct diagnosis of actinomycetoma secondary to *Nocardia species* infection was subsequently made. This was only possible with evidence from the re‐biopsy showing the Gram‐positive filamentous bacteria, not initially observed with the first biopsy. In light of this and in keeping with the Neafie and Marty proposal,^11^ a diagnosis of cutaneous botryomycosis was unsupported due to evidence of the filamentous bacteria. Unexpectedly, a hematoma was detected up on USS of the thigh, suggestive of the rarely observed vascular damage due to actinomycetoma.^12^ This observed hematoma explains the persistent microcytic anemia which incidentally started resolving on the commencement of appropriate antibiotic therapy for actinomycetoma. Thus, potentially indicating the cessation of the actinomycetoma associated vascular destruction.

Similar cases of an initial diagnosis of cutaneous botryomycosis later revealing laboratory findings suggestive of actinomycetoma have previously been reported.^13^, ^14^ In a case report from Pal et al., an initial diagnosis of cutaneous botryomycosis was revised to actinomycetoma following lack of response to a week's course of antibiotics and the subsequent observation of filamentous bacteria and the Splendori–Hoeppli reaction on histological examination.^14^ The patient subsequently showed significant improvement 2 months into a 6‐month course of amoxycillin and clavulinic acid 1 g twice daily.^14^ In another case report, DeWitt et al present a case of cutaneous botryomycosis due to *MRSA* which responded poorly to surgical debridement and multiple antibiotics inclusive of ceftriaxone.^13^ Clinical response was only observed after starting the patient on linezolid and minocycline with subsequent biopsy findings culturing *Norcadia Mexicana* from the same wounds.^13^ The latter findings suggest a diagnosis of actinomycetoma since it responds to both linezolid and minocycline.^15^ However, despite culturing *Norcadia Mexicana* and its suggestive therapeutic response, the authors did not document a revision of their diagnosis to actinomycetoma. Likewise, several cases of cutaneous botryomycosis have also initially been misdiagnosed as actinomycetoma only for the diagnosis to be revised following failure to respond to appropriate therapy for the latter.^7^, ^16^ These four cases clearly demonstrate the well characterized clinical and histopathological mimicry of cutaneous botryomycosis to actinomycetoma, thus confounding disease diagnosis.^7^ Furthermore, they indicate that failure to respond to the indicated antibiotic therapy for the suspected clinical diagnosis should prompt a re‐evaluation of the clinical and investigation findings.

The Splendori–Hoeppli phenomenon describes a histopathologic appearance of asteroid, radiating, intensely eosinophilic material around infectious organisms or biologically inert substances.^6^ In bacterial and fungal infections, it forms a peripheral cuff surrounding the organism whereas in non‐infectious conditions, it appears as a necrotic center within a granuloma surrounded by eosinophils, epithelioid histiocytes, lymphocytes, and multinucleated giant cells.^6^ It is a useful finding in a clinical presentation such as our patient's as it helps eliminate clinical differentials such as Kaposi sarcoma. However, the occurrence of this reaction pattern obscured the actual diagnosis as our clinical differential diagnoses also included histopathologic differentials of the Splendori–Hoeppli reaction.^6^ The diagnosis was eventually clinched by performing a deeper biopsy, additional stains and re‐doing the gram stain. We observed Gram‐positive, filamentous rods in addition to the absence of staining with either Ziehl Nielsen or Fite stains. This strongly suggested *Nocardia species* infection and a diagnosis of actinomycetoma.^6^, ^11^ Furthermore, the nasal swab which cultured *MRSA* indicated nasal carriage of *MRSA*, which was managed by prescribing topical mupirocin. Unfortunately, no further nasal swabs were done subsequently to check clearance of the nasal carriage.

There are several lessons in this case presentation. Firstly, failure of the infection to respond to the recommended antibiotic therapy should prompt a re‐evaluation of the diagnosis. Even though both cutaneous botryomycosis and actinomycetoma respond to cotrimoxazole,^9^, ^11^, ^17^ the initial failure of the suspected cutaneous botryomycosis potentially indicated an erroneous diagnosis or resistance to therapy. Actinomycetoma tends to respond later and on use of combination therapy of cotrimoxazole with amikacin (Welsh regimen) or amikacin and rifampicin (Modified Welsh regimen) or gentamycin and doxycycline (Modified two‐step regimen).^17^ Furthermore, the continued failure of the *MRSA* sensitivity directed vancomycin therapy rightfully prompted a re‐evaluation of the clinical diagnosis.^11^ Secondly, in difficult cases, constituting a multi‐disciplinary team assists in obtaining the correct diagnosis and treatment plan for the patient. Clinico‐pathologic correlation involving both the dermatologist and the histopathologist is crucial to make an accurate diagnosis in granulomatous cutaneous dermatoses.^18^ Finally, re‐biopsing may improve the pickup of the causative organism. In our case, the *Nocardia spp* was only observed on the re‐look biopsy as it had been missed on the initial biopsy. This could be due to small numbers of viable organisms present in longstanding lesions such as these in our patient or inadequate material on the specimen among other reasons.^17^ Repeat biopsies and strong collaboration of dermatologists, medical microbiologists, and histopathologists potentially increases the chances of getting to an accurate histopathological diagnosis.^19^

## 6 CONCLUSION

The presented case serves as a reminder of the diagnostic challenges posed by rare infectious diseases and the utility of constituting multi‐disciplinary teams in their management. Furthermore, a thorough understanding of clinico‐pathologic nuances is crucial to prevent misdiagnosis and ensure timely and effective management especially for inflammatory dermatoses.^20^

## AUTHOR CONTRIBUTIONS

**Josiah Tatenda Masuka:** Conceptualization; formal analysis; investigation; methodology; resources; visualization; writing – original draft; writing – review and editing. **Luanda Mthembu:** Data curation; resources; writing – review and editing. **Khumo Duze:** Investigation; resources; writing – review and editing. **Ameshin Moodley:** Conceptualization; investigation; resources; writing – review and editing. **Tshikani Norman Rikhotso:** Conceptualization; investigation; methodology; resources; supervision; validation; visualization; writing – review and editing. **Anisa Mosam:** Conceptualization; investigation; methodology; resources; supervision; visualization; writing – review and editing.

## FUNDING INFORMATION

This research received no specific grant from any funding agency in the public, commercial, or not‐for‐profit sectors.

## CONFLICT OF INTEREST STATEMENT

All authors declare that they have no competing interests.

## CONSENT

Written informed consent was obtained from the patient to publish this report in accordance with the journal's patient consent policy.

## Data Availability

The data that support the findings of this study are available from the corresponding author upon reasonable request.
